# Continuous Synthesis of 2‐Methoxyhydroquinone from Vanillin in a Taylor‐Couette Disc Contactor

**DOI:** 10.1002/cssc.202501042

**Published:** 2025-09-16

**Authors:** Annika Grafschafter, Georg Rudelstorfer, Dominik Wickenhauser, Christian Leypold, Werner Schlemmer, Stefan Spirk, Susanne Lux

**Affiliations:** ^1^ Institute of Chemical Engineering and Environmental Technology Graz University of Technology Inffeldgasse 25C 8010 Graz Austria; ^2^ Institute of Bioproducts and Paper Technology Graz University of Technology Inffeldgasse 23 8010 Graz Austria

**Keywords:** 2‐methoxyhydroquinone, redox flow batteries, Taylor‐Couette disc contactor, vanillin

## Abstract

Organic redox flow batteries are a promising sustainable technology for large‐scale storage of surplus energy in the grid. However, the main challenge lies in scaling up the synthesis of redox‐active molecules from sustainable sources, as either the synthesis is complex or renewable feedstock is not available at a large scale. This challenge is addressed by employing vanillin, a fine chemical widely available from lignin, as a starting material for the synthesis of redox‐active hydroquinones. The use of a continuously operating column reactor, known as the Taylor‐Couette disc contactor, is demonstrated to generate redox‐active 2‐methoxyhydroquinone in high yields and purity. Production rates of ≈0.55 kg h^−1^ of 2‐methoxyhydroquinone are achieved, with sufficient purity for use in single‐cell redox flow battery performance and short‐stack stability tests.

## Graphical abstract

This study investigates continuous synthesis of 2‐methoxyhydroquinone (MHQ) from vanillin in a Taylor‐Couette disc contactor (TCDC). The TCDC, a stirred column reactor utilizing Taylor vortices for intense mixing, achieved efficient phase separation and hydraulic stability. With an 83% yield, 93.8% purity, and a production rate of 0.55 kg h^−1^ of MHQ, the product proves suitability for redox flow batteries.

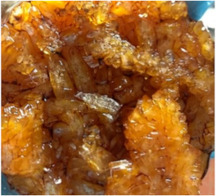

## 1 Introduction

Energy storage plays a crucial role in modern energy systems, especially as the world transitions to renewable energy sources like wind and solar power. These sources are intermittent, producing energy only when conditions are favorable, making efficient storage systems essential to ensure a steady power supply. For instance, pumped hydroelectricity can store significant amounts of energy at a low cost and currently accounts for 98% of the global energy storage. Alternatively, lithium‐ion batteries are the second‐largest energy storage technology.^[^ ^1^ ^]^ However, the potential sites for new pumped storage hydropower systems are limited due to the specific geographical requirements. Moreover, lithium‐ion batteries have a significant environmental impact, and recycling is still a challenging task.^[^ ^1^ ^]^

Among the various storage technologies, redox‐flow batteries (RFBs) stand out for their scalability, long cycle life, and ability to store large amounts of energy. Energy is stored and converted through a chemical reaction, with electrical energy being stored in the electrolyte based on the oxidation state of the redox‐active molecule in the individual cells. The storage capacity of an RFB directly depends on the quantity of the redox‐active material, and electrical power can be independently scaled by increasing the number of individual cells. This makes them ideal for grid‐scale applications and renewable energy integration. RFBs are also safer than many other battery types, as they operate at lower risks of thermal runaway.^[^ ^2^ ^]^

State‐of‐the‐art RFBs typically use vanadium, which is extracted from ores through energy‐intensive mining and refining processes, raising environmental and economic concerns.^[^ ^3^ ^]^ Organic molecules represent an environmentally friendly alternative to vanadium and are of major interest in the scientific community.^[^ ^4^ ^]^ Quinones, which can be produced from biobased materials, are promising candidates as electroactive molecules in RFBs. Another key raw material available in large quantities is lignin, produced in the paper and pulp industry at over 50 million tons per year. Vanillin, one of the few chemicals produced at a significant scale from lignin, currently accounts for about 3,000 tons annually, which represents ≈15% of global vanillin production.^[^ ^5^ ^]^ Vanillin has been suggested as a stable, lignin‐based product for the green synthesis of 2‐methoxyhydroquinone (2‐methoxy‐1,4‐benzenediol, MHQ) using hydrogen peroxide as a convenient oxidant.^[^ ^6^ ^]^ However, conventional MHQ synthesis methods often involve multistep batch processes, harsh reaction conditions, and the use of toxic reagents, resulting in limited efficiency and scalability. Recently, the reaction has been modified to achieve shorter reaction times under ambient conditions by employing tetrahydrofuran (THF) as a solvent to increase vanillin solubility, as vanillin has very limited solubility in water (10 g L^−1^ at 25 °C). Detailed information on the use of THF as a solvent in this context is provided.^[^ ^7^ ^]^ The reaction yield at higher vanillin concentrations and the efficiency of product isolation can be improved by using solid sodium percarbonate as a convenient hydrogen peroxide source. Commonly employed as a bleaching agent in the detergent industry, sodium percarbonate is inexpensive and readily available on a large scale. The general reaction mechanism for the conversion of vanillin to MHQ is illustrated in Figure 4. The reaction proceeds via nucleophilic addition of the hydroperoxide anion, present under basic conditions, to the aldehyde carbonyl group. Formic acid is formed as a by‐product, leading to acidification of the reaction mixture. According to Fache et al.,^[^ ^6^ ^]^ final acidification of the reaction mixture is crucial, as under acidic conditions, carbonates and hydroperoxide anions are no longer present in solution, effectively stopping the reaction. However, excessively acidic conditions due to high acetic acid concentrations have a negative effect on MHQ concentration. In this regard, sodium percarbonate offers a clear advantage over aqueous hydrogen peroxide (H_2_O_2_), as it provides excellent buffer capacity for pH stabilization. Additionally, combining sodium percarbonate with vanillin in a THF‐solvent phase offers the advantageous side effect of phase separation, facilitated by the salting‐out effect of sodium carbonate. The salting‐out effect is based on the addition of a substance (sodium percarbonate) to a water‐miscible organic solvent (THF), inducing phase separation from the remaining aqueous solution due to altering its ionic strength. Consequently, it employs the spontaneous formation of a biphasic system and simultaneous extraction of a target compound (MHQ) into a separated organic solvent (THF)‐rich phase. Thus, the product, MHQ, is primarily dissolved in the solvent phase and can be easily isolated through solvent evaporation. Sodium carbonate also counteracts the acidification caused by formic acid, resulting in the formation of CO_2_. Initially, the system contains two miscible phases, which, upon mixing and CO_2_ evolution, undergo phase separation driven by salting‐out.

Continuous MHQ production under these conditions requires an advanced reactor design capable of efficient mixing, phase separation, and handling of gas‐phase formation. While stirred tank reactors provide good mixing, they suffer from large reactor volumes, higher capital costs, and broad residence time distributions, which can negatively affect product quality and process efficiency. Tubular reactors, although more compact and cost‐effective, typically operate at lower hydraulic loads and are less suitable for handling complex multiphase flow. In particular, gas formation poses a significant challenge in conventional tubular reactor setups. To ensure industrial applicability, the synthesis must be scalable and suitable for continuous operation. For this purpose, the Taylor‐Couette disc contactor (TCDC) was selected, an innovative, stirred extraction column capable of handling continuous multiphase flow, including systems involving up to four phases simultaneously. The conversion of vanillin to MHQ via Dakin oxidation was first optimized in batch experiments and subsequently implemented in the TCDC to demonstrate its feasibility for continuous processing.

## 2 Results and Discussion

### 2.1 Batch Experiments

The reaction proceeded efficiently under the established alkaline conditions, with sodium percarbonate serving as a convenient and stable hydrogen peroxide source. Maintaining a constant phase ratio of aqueous to organic phase at 2 enabled higher vanillin concentrations without limiting oxidant availability, as hydrogen peroxide was supplied in excess (≥5 equivalents). Upon increasing reaction progress, a phase separation occurs due to the better solubility of MHQ in a THF‐rich phase.

**Figure 1:**
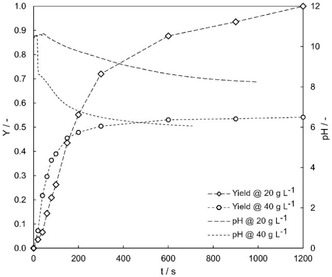
Yield of MHQ over time for initial concentrations of 20 g L^−1^ vanillin in THF (26 g L^−1^ H_2_O_2_ (0.51 mol L^−1^) in 0.1 M NaOH solution) and 40 g L^−1^ vanillin in THF (26 g L^−1^ H_2_O_2_ (0.51 mol L^−1^) in 0.1 M NaOH solution), with an aqueous‐to‐solvent phase ratio of 2 and a constant temperature of 30 °C. The corresponding pH values are shown on the secondary y‐axis as dashed lines without markers.

It was observed that the MHQ yield decreases with increasing reactant concentration, as shown in **Figure** **1** for initial vanillin concentrations of 20 g L^−1^ (0.0439 mol L^−1^) and 40 g L^−1^ (0.0877 mol L^−1^). Limited yield and conversion are observed at higher vanillin concentrations due to a greater amount of formic acid formed from the cleaved carboxyl group, which significantly lowers the pH at the reaction's outset. A fast decrease in the reaction rate of Dakin oxidation at lower pH values has also been reported by Hocking et al.^[^ ^8^ ^]^ Alkaline conditions are crucial for increasing yield and conversion. The yield of MHQ at higher vanillin concentrations can be enhanced by using sodium percarbonate as a hydrogen peroxide source to counteract rapid acidification. An increase in the NaOH concentration does not yield the same result, as this increases the rate of decomposition of formed MHQ in the aqueous phase and at the phase contact areas. Using sodium percarbonate benefits phase separation after reaction, with the product being dissolved in the solvent phase, simplifying the final product isolation through solvent evaporation and subsequent crystallization.

### 2.2 Reaction Enthalpy

The synthesis of MHQ from vanillin through Dakin oxidation is a highly exothermic reaction, making the determination of reaction enthalpy essential for scaling up the process to ensure effective cooling. The reaction enthalpy was measured under isolated conditions at two different concentrations, while maintaining a constant hydrogen peroxide‐to‐vanillin ratio.

The amount of energy was subtracted from the measured reaction enthalpy. To calculate the reaction enthalpy, the initial reaction temperature (*T*_initial_) and the maximum temperature (*T*_max_) reached were determined from the temperature curves, incorporating the blank measurement data. Using these two temperatures, the reaction enthalpy for the conversion of vanillin to MHQ was calculated with Equation (1). An ideal liquid with a constant heat capacity was assumed, and the effects of NaOH, sodium percarbonate, and vanillin on heat capacities were considered negligible. The heat capacity of water at 25 °C is given as *Cp*_H2O_ = 4.182 J g^−1^ K^−1^ and for THF as *Cp*_THF_ = 1.72 J g^−1^ K^−1^.^[^ ^9^, ^10^ ^]^

(1) $$\Delta_{R}H_{m}=\frac{\left(m_{H_{2}O}\cdot Cp_{H_{2}O}+m_{\text{THF}}\cdot Cp_{\text{THF}}\right)\cdot \left(T_{\text{initial}}-T_{\text{max}}\right)}{\frac{m_{\text{Vanillin}}}{M_{\text{Vanillin}}}}$$

The reaction enthalpy of Δ_R_ *H*_m_ = −612.49 in kJ mol^−1^ was determined from 14 measurements with a standard deviation of 4.25 kJ mol^−1^. The result emphasizes the importance of thermal regulation in both laboratory and industrial setups.

### 2.3 Residence Time Distribution (RTD)

The RTD of the TCDC column was investigated to assess deviations from ideal plug‐flow behavior, which are important for column design.

**Figure 2:**
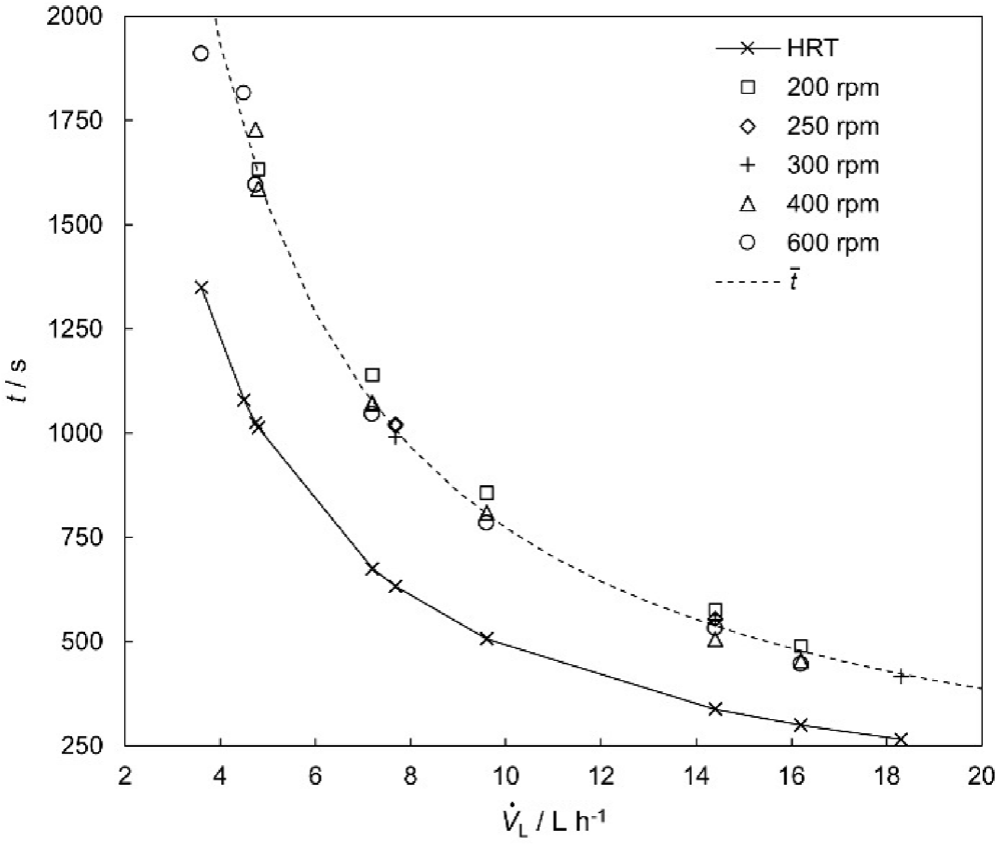
Influence of the volumetric flow rate and rotational speed on the measured residence time. The mean residence time $\bar{t}$ is depicted as a dotted line. The HRT is shown as a solid line.

**Figure** **2** illustrates the influence of both the volumetric flow rate ${\dot{V}}_{L}$ and rotational speed on the RTD and the corresponding hydraulic residence time (HRT). The rotational speed hardly influences the residence time under cocurrent flow conditions. With increasing ${\dot{V}}_{L}$ the HRT and the mean residence time $\left(\bar{t}\right)$ decrease.

**Figure 3:**
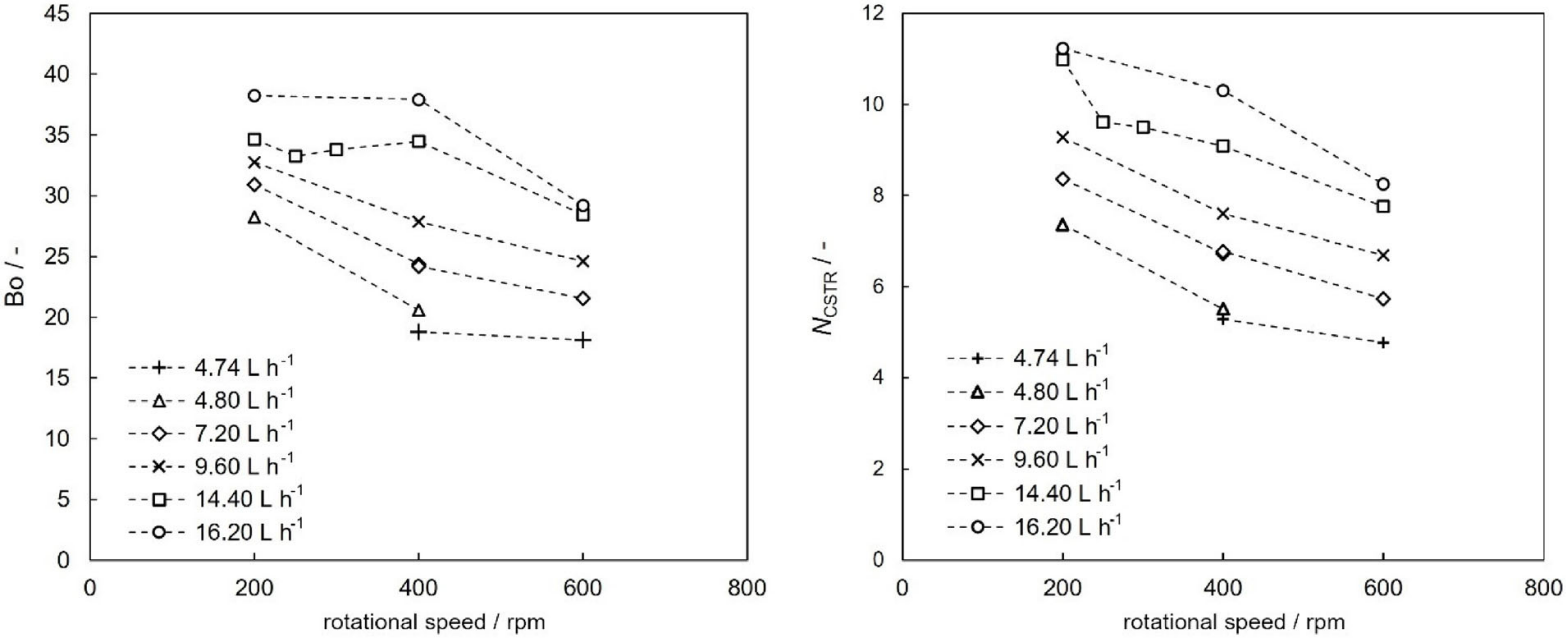
Effect of rotational speed and liquid phase flow rate on Bodenstein number (left) and number of CSTRs in series (right).

The results of the evaluation of the Bo‐number and the corresponding number of vessels in series (*N*_CSTR_) at different rotational speeds and volumetric flow rates are presented in **Figure** **3**. Large deviation from plug flow is indicated by Bo < 100, suggesting application of the continuous stirred tank reactor (CSTR) model for RTD analysis. Axial backmixing increases (Bo and *N*_CSTR_ decrease) with higher rotational speed due to increased axial dispersion, while it decreases with higher flow rates (Bo and *N*_CSTR_ increase) due to enhanced plug flow behavior. The investigated TCDC column consists of 33 compartments, which are equivalent to 5–11 *N*_CSTR_.

### 2.4 Continuous Experiments Using the TCDC

Continuous MHQ production requires an advanced reactor design concept capable of efficient mixing, phase separation, and handling of gas‐phase formation. For this purpose, the TCDC was selected. This column‐type stirred contactor was originally developed for liquid/liquid extraction and is designed with inherent separation zones, allowing for the lighter solvent phase to rise to the top and the heavier aqueous phase to settle at the bottom.^[^ ^11^, ^12^ ^]^ Recent applications of the TCDC for continuous gas/liquid flow^[^ ^13^ ^]^ have demonstrated its effectiveness in handling multiphase systems, enabled by the simple design of internals and the use of perforated rotor discs. Before initiating continuous experiments, a series of batch experiments was conducted at a 1‐liter scale in a double‐jacketed stirred glass reactor to evaluate phase separation behavior and product quality. The results indicated that a vanillin concentration of 200 g L^−1^ in THF and 150 g L^−1^ sodium percarbonate in a 0.1 M NaOH solution are recommended for continuous experiments, as they provide a sufficient excess of hydrogen peroxide (2.17 equivalents) to ensure complete conversion of vanillin.

The reaction yield at higher concentrations and the efficiency of product isolation were improved by using solid sodium percarbonate as a convenient hydrogen peroxide source. When sodium percarbonate is combined with vanillin in a THF‐based solvent system, phase separation occurs as a beneficial side effect, driven by the salting‐out effect of the resulting sodium carbonate. MHQ preferentially dissolves in the THF‐rich organic phase, allowing for straightforward isolation via solvent evaporation. The sodium carbonate also counteracts the acidification caused by formic acid, releasing CO_2_ in the process. Initially, the system comprises two miscible phases that, upon mixing and concurrent gas evolution, separate due to both the salting‐out effect and the preferential solubility of MHQ in the THF‐rich layer. This also protects freshly formed MHQ from degradation, which occurs within seconds in aqueous alkaline media.

**Figure 4:**
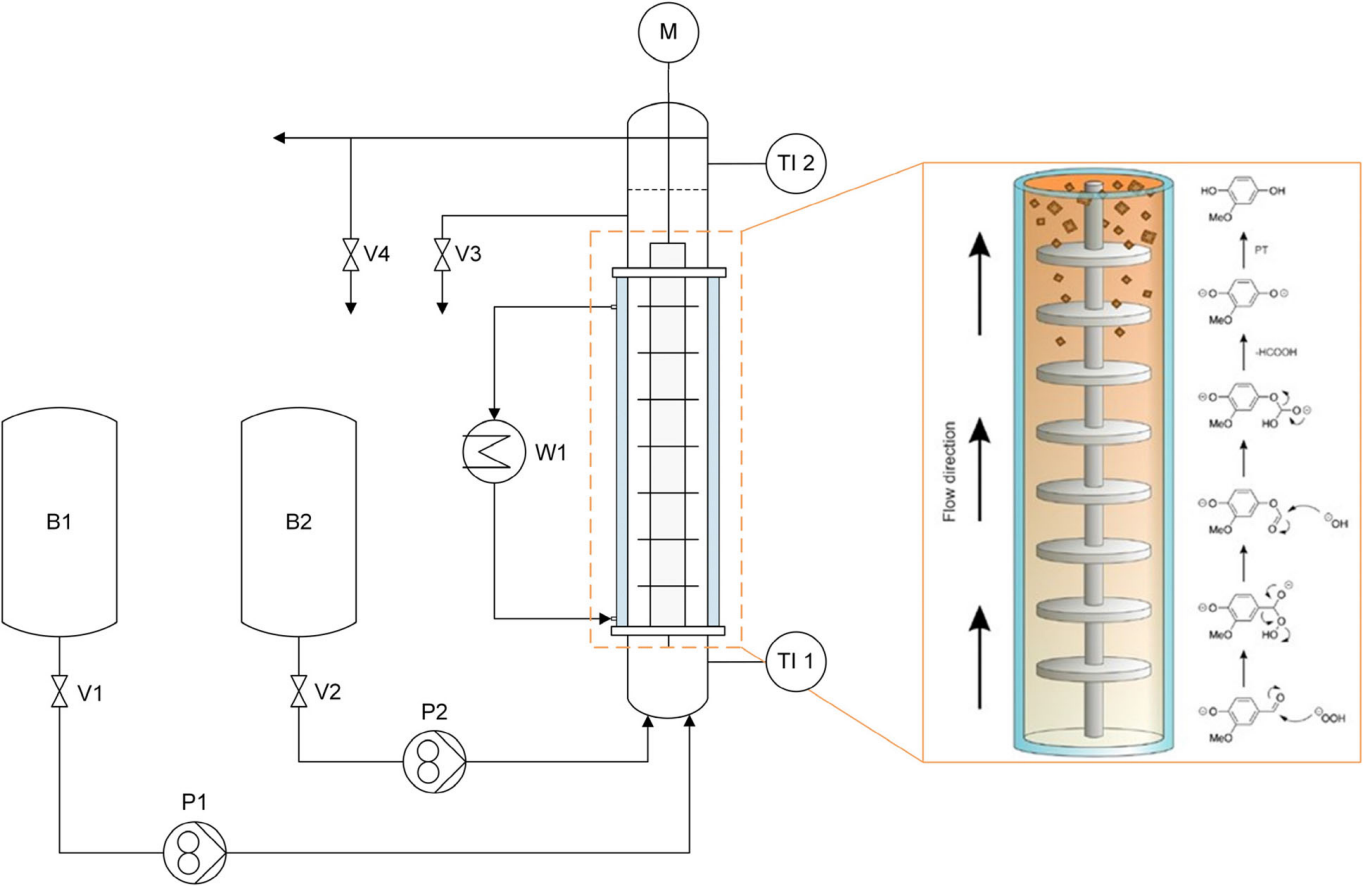
Schematics of the TDCD setup (B1: THF phase containing vanillin; B2: aqueous NaOH phase containing dissolved sodium percarbonate) and the reaction mechanism along the flow direction and column height, showing increasing phase separation toward the upper section.

Continuous MHQ synthesis requires the cocurrent operation of the TCDC column. Both phases—the vanillin‐containing solvent phase and the oxidant‐containing aqueous phase—are fed at the bottom of the column in the desired phase ratio and flow rates. The two feed tanks (B1 and B2 in **Figure** **4**) are used to store the reactant‐containing solvent and aqueous phases. The startup of the column for continuous MHQ synthesis began by feeding the sodium percarbonate solution containing 0.1 M NaOH until the liquid phase reached the first rotor disc of the TCDC column. Next, the vanillin‐containing solvent phase was added, and the stirrer was activated. The reaction started immediately, as evidenced visually by a color change from the transparent initial solution over yellow to a red–brown hue in the reaction broth. As the liquid level rose at the desired flow rate, the aqueous phase outlet valve (V3 in Figure 4) was kept closed until the two phases started to separate. The phase interface between the MHQ‐containing solvent phase and the aqueous phase at the top of the column was then adjusted by carefully opening valve V3 and fine‐tuning its position until steady‐state operation was achieved. The phase separation was visually indicated when the reaction mixture turned cloudy.

The reaction mechanism, as depicted in Figure 4, takes place along the column height. Taylor vortices, which arise between the rotor discs after exceeding a certain rotational speed,^[^ ^11^, ^12^, ^13^ ^]^ ensure distinct mixing of the reactants throughout the entire reaction zone. At a specific column height, the solvent and aqueous phases begin to separate, with the solvent‐rich phase on top of the aqueous one. A phase interface between the MHQ‐containing solvent phase and the aqueous phase is formed in the settling zone at the top of the column.

Steady‐state process conditions are summarized in **Table** **1**, including the results from the RTD evaluation. In initial long‐term experiments, consistent product quality was maintained over 5 h of steady‐state operation, with no clogging observed in the reactor. The water content in the solvent phase was measured to be 18.2 wt%, necessitating water removal by evaporation after solvent removal via evaporation. This would enable solvent recycling if potential loss of peroxide formation inhibitors present in the solvent is compensated. The remaining product, a dark brown liquid, was placed in crystallization beakers and stored in a vacuum drying oven at 50 °C and 100 mbar for two days. The resulting yellow crystals were milled into powder and used in redox flow cells for characterization and stability tests.^[^ ^7^ ^]^ Steady‐state continuous synthesis resulted in an 83% product yield (*Y*_MHQ_), with a steady‐state production rate of 0.55 kg h^−1^ of MHQ. This value was obtained from concentration measurements at the solvent phase outlet and corresponds to the weighted amount of crystalline product. Samples were taken during the entire experiment and were analyzed by HPLC. The relative conversion of vanillin (*X*_Vanillin_) at steady state was 97%, with the remaining vanillin dissolved in the aqueous phase. Additionally, 3% of the MHQ product likely degraded within the aqueous phase.

**Table 1:** Operation conditions of continuous MHQ synthesis from vanillin at steady‐state operation.

Name	Value	Unit
$\dot{V}$_aq_ (aqueous feed)	0.12	L min^−1^
*c*_aq,NaOH,in_	40	g L^−1^
*c*_aq,Per,in_ (active peroxide)	150	g L^−1^
*c*_aq,Van,out_	0.4	g L^−1^
*c*_aq,MHQ,out_	2.5	g L^−1^
$\dot{V}$_solvent_ (solvent feed)	0.06	L min^−1^
*c*_sol,Van,in_	200	g L^−1^
*c*_sol,Van,out_	0	g L^−1^
*c*_sol,MHQ,out_	154	g L^−1^
*m*_sol,H2O,out_	18.2	wt%
*Y*_MHQ_	83	%
*X*_Vanillin_	97	%
*n*	200	rpm
*N*_CSTR_	9	–
Bo	32.7	–
Dax	5.8 E‐05	m^2^ s^−1^
HRT	506.2	s
$\bar{t}$	857	s
*T*_in_	25	°C
*T*_out_	29	°C

**Figure 5:**
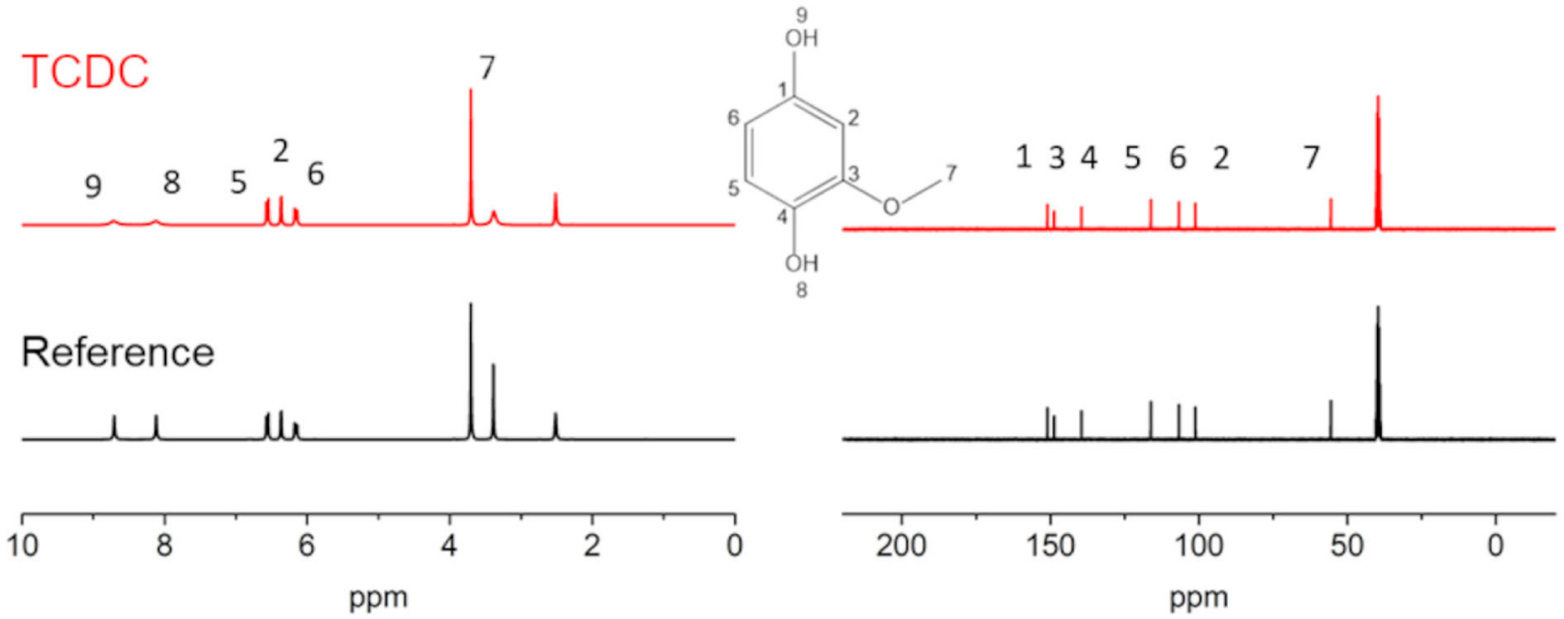
^1^H (left) and ^13^C (right) NMR of a MHQ product reference (black, bottom) and the crystallized reactor product (red, top). ^1^H NMR (300 MHz, DMSO‐d6, ppm) *δ* 3.73 (s, 3 H, H7), 6.18 (dd, 3J5,6 = 8.40 Hz, 4J6,2 = 2.65 Hz, 1 H, H6), 6.39 (d, 4J7,8 = 2.65, 1 H, H2) (d, 3J5,6 = 8.42 Hz, 1 H, H5) 8.14 (s, 1 H, H8), 8.73 (s, 1 H, H9). ^13^C NMR (300 MHz, DMSO‐d6, ppm) *δ* 55.39 (s, C7), 100.74 (s, C2), 106.23 (s, C6), 115.69 (s, C5), 138.88 (s, C4), 148.06 (s, C3), 150.30 (s, C1).

The crystalline product was analyzed by nuclear magnetic resonance (NMR) spectroscopy and compared with a purchased reference sample as listed in Table S1 in the Supporting Information. The ^1^H and ^13^C spectra are exemplarily shown in **Figure** **5**. The purity of the produced MHQ was also measured using GC‐MS, yielding a result of 93.8%. Further purification by sublimation is possible but not necessary for application as electrochemically active compound in organic RFBs.

## 3 Conclusion

The scale‐up of the continuous synthesis of a redox‐active molecule, MHQ, from vanillin, a biobased feedstock, was demonstrated. The TCDC reactor was shown to be highly suitable for performing such reactions, enabling tailored synthesis and separation while being easily scalable, either by parallelization or by using larger and wider columns. Determining reaction parameters, such as reaction enthalpy and kinetics in a batch process, is crucial for seamless adaptation to a continuously operated reactor like the TCDC. In addition to the scale‐up, synthesis efficiency was improved by using percarbonate as an oxidizing agent, enabling quantitative conversion to MHQ. Although the product was isolated in high yields, further improvements are required to develop a fully commercial, sustainable process. The use of more sustainable solvents, such as 2‐methyl‐THF, identified through safe‐and‐sustainable‐by‐design studies, presents a promising alternative to THF.^[^ ^14^ ^]^ 2‐Methyl‐THF is not only less toxic,^[^ ^15^ ^]^ but also represents a greener alternative compared to THF, as it can be sourced from renewable resources leading to reduced emission (by 97%,^[^ ^16^ ^]^). The better drying properties of 2‐methyl‐THF over THF facilitate quantitative recovery of 2‐methyl‐THF after phase separation. Circularity of the used solvent is a clear economic benefit. Moreover, the higher boiling point of 2‐methyl‐THF (80 °C) compared to THF (64 °C) allows for a larger reaction window and thus enhances the safety of the process. Initial trials with the sustainable solvent 2‐methyl‐THF indicated that the batch process follows similar kinetics and yields and comparable product quality. A detailed study is still pending and was not within the scope of this study. However, the main challenge of the overall synthesis route still lies in designing a continuous product isolation process, which will likely involve sublimation or spray drying. These methods are currently under investigation in the lab. Furthermore, isolating the by‐product acetic acid from the aqueous effluent remains a key step to be addressed.

In conclusion, the use of the Taylor‐Couette Disc Contactor (TCDC) for continuous MHQ synthesis from vanillin provides several advantages over conventional reactor designs. Compared to stirred tank reactors, which are typically associated with large reactor volumes, high capital costs, and broad residence time distributions, the TCDC offers a more compact footprint, enhanced mixing efficiency, and narrower residence time distributions. In contrast to tubular reactors or microreactors, which are generally limited by lower hydraulic loads, the TCDC can handle significantly higher throughputs, making it highly suitable for scale‐up and continuous industrial operation. Additionally, the TCDC excels in managing complex multiphase flow, accommodating up to four phases simultaneously while ensuring effective mixing and separation within a single column setup.

## 4 Experimental Section

The chemicals used in this study are listed in Table S1 in the Supporting Information.

4.1

4.1.1

##### Batch Experiments

Reaction kinetics were investigated using a custom‐made mini reactor made of stainless steel with an inner diameter of 12 mm and an inner tube height of 30 mm. The wall thickness was 5 mm. A thermocouple (type K), installed in a 1.5 mm hole, was used to measure the temperature of the reaction mixture. The temperature data were recorded using a custom‐built, Arduino‐based data acquisition (DAQ) unit. During the experiments, the reactor was placed in a temperature‐controlled water bath (Lauda RC20) to maintain isothermal conditions. Additionally, the reaction mixtures were precooled to prevent a sudden temperature increase due to mixing enthalpy. Intense mixing within the reactor was achieved using a small magnetic stirring bar.

For batch experiments, the phase ratio of the hydrogen peroxide‐containing aqueous phase to the vanillin‐containing solvent phase was kept constant at 2. This optimized approach allows for increased vanillin concentration and ensures sufficient hydrogen peroxide capacity in the aqueous phase for highly concentrated reactions. Hydrogen peroxide was added in excess (≥5 equivalents) to prevent conversion limitations due to oxidant depletion.

A separate experiment was conducted for each data point, with the reaction broth being quenched by acidification with acetic acid after the desired time. For the experiments, 1 mL aqueous phase was mixed with 0.5 mL organic phase and quenched with 0.2 mL of concentrated acetic acid. pH levels were recorded in a glass beaker (containing 20 mL of the organic phase and 40 mL of the aqueous phase) under identical concentrations and temperatures.

The concentrations of vanillin and MHQ were determined by high‐performance liquid chromatography (HPLC), with further details provided in the Supporting Information. Each experiment was performed twice, and the average concentrations were used to calculate yield and conversion.

##### Reaction Enthalpy

The determination of the reaction enthalpy was conducted in an isolated, closed Dewar vessel. The temperature increase over time was monitored using a custom MATLAB routine with a type K thermocouple. Constant mixing of the reaction mixture was maintained using a laboratory stirrer (WiseStir HT 50 DX, 50–1000 rpm) operating at low rotational speed.

Two different concentrations, maintaining a constant hydrogen peroxide‐to‐vanillin ratio, were used for the determination of the reaction enthalpy. To prevent temperature changes caused by solvation processes, the solvent and aqueous phases were prepared outside the reaction vessel. For each measurement, 200 mL of 0.1 M NaOH solution containing 44 or 88 g L^−1^ sodium percarbonate was first added to the Dewar vessel. The stirrer was then activated, followed by the addition of 100 mL of vanillin‐containing THF solution (40 or 80 g L^−1^). Finally, the vessel was sealed with a plastic lid. The temperature change due to the enthalpy of mixing was considered by performing blank measurements, replicating the experiments without vanillin in the THF solution.

##### RTD

Hydraulic characterization was performed by measuring the RTD using deionized water as the continuous liquid phase. A pulse of saturated sodium chloride solution was injected at the bottom of the column, and conductivity changes were monitored at the outlet of the active mixing zone. These nonreactive tracer experiments evaluated the influence of rotational speed and volumetric flow rates on the RTD. The normalized conductivity data were interpreted using a combination of CSTR cascade and axial dispersion models. Detailed descriptions of the mathematical analyses, including the relevant equations for the dimensionless exit age distribution (*E*_*θ*_), Bodenstein number (Bo), and axial dispersion coefficient (*D*_ax_), are provided in the Supporting Information. The HRT was determined based on the reactor volume (*V*_Reactor_) and volumetric flow rate of the liquid (${\dot{V}}_{L}$) using Equation (2).

(2) $$\text{HRT}=\frac{V_{\text{Reactor}}}{\dot{V}L}$$

##### Continuous Experiments

For the continuous experiments, a TCDC was used with the geometric dimensions provided in the Supporting Information (Table S2, Supporting Information). The piping and instrumentation diagram (P&ID) for the MHQ synthesis performed in the TCDC is shown in Figure 4, where the main components are labeled.

The two individual gear pumps (Ismatec–Reglo Z with MICROPUMP GJ‐N21.FF2S.B pump head for the aqueous phase and GA‐X21.CFS.B pump head for the solvent phase) are installed to maintain a constant flow rate of both phases. The temperature of the reaction mixture is measured before and after the active mixing height using a thermocouple type K (TI 1 and TI 2). A double‐jacketed glass column connected to a heat exchanger (MGW Lauda ‐ Kryomat RUK 40 S) is used to provide adequate cooling (W1). The rotor is powered by a laboratory stirrer (WiseStir HT 50 DX, 50–1000 rpm). The control valve V3 is used to adjust the flow rate of the heavy aqueous outlet and to set the phase interface in the top settling zone. V4 remains permanently open to ensure an unhindered flow of the product‐containing solvent phase. The two feed tanks (B1 and B2) are used to store the reactant‐containing solvent and aqueous phases.

The crystalline product was analyzed by NMR and compared with a purchased reference sample as listed in Table S1 in the Supporting Information. A Bruker 300 MHz NMR instrument was used to acquire ^1^H and ^13^C spectra. For the analysis, 20 mg of the sample was dissolved in DMSO‐d6, and the signals were referenced by the DMSO signal at 2.50 ppm (^1^H) and 39.52 ppm (^13^C). The spectra were averaged over 16 scans with a 1‐second delay, and the data were processed using Mestrenova software from Mestrelab.

## Supporting Information

The authors have cited additional references within the Supporting Information.^[^ ^17^ ^]^

## Conflict of Interest

The authors declare no conflict of interest.

## Supporting information

Supplementary Material

## Data Availability

The data that support the findings of this study are available from the corresponding author upon reasonable request.
