# Supplementary material for: Continuous Synthesis of 2‐Methoxyhydroquinone from Vanillin in a Taylor‐Couette Disc Contactor
Source: ChemSusChem. 2025 Sep 16;18(20):e202501042. doi: 10.1002/cssc.202501042 (PMC12548935; doi:10.1002/cssc.202501042)
Supplement: Supplementary file 1 — Supplementary Material [file CSSC-18-e202501042-s001.pdf]

Supporting information

Continuous synthesis of 2-methoxyhydroquinone from vanillin in a Taylor-Couette Disc Contactor

Annika Grafschafter*[a], Georg Rudelstorfer[a], Dominik Wickenhauser[b], Christian Leypold[a], Werner Schlemmer[b], Stefan Spirk[b] and Susanne Lux[a]

[a] Dr. techn. A. Grafschafter, Dr.techn. G. Rudelstorfer, Dr. techn. C. Leypold, and Assoc.Prof. Dipl.-Ing. Dr.techn. S. Lux
Institute of Chemical Engineering and Environmental Technology
Graz University of Technology
Inffeldgasse 25C, 8010 Graz, Austria
E-mail: a.grafschafter@tugraz.at

[b] Dipl.-Ing. D. Wickenhauser, Dr. techn. W. Schlemmer and Assoc.Prof. Mag.rer.nat. Dr.rer.nat. S. Spirk
Institute of Bioproducts and Paper Technology
Graz University of Technology
Inffeldgasse 23, 8010 Graz, Austria

Table of Contents

1. Materials and Methods 2

1.1 Chemicals used 2

1.2 Batch experiments 2

1.3 Reaction enthalpy 3

1.4 Continuous experiments 4

1.5 Residence time distribution 6

1.6 High performance liquid chromatography 7

1.7 Nuclear Magnetic Resonance 7

1. Materials and Methods

1.1 Chemicals used

The chemicals used in this study are listed in Table S1.

Table S1. Chemicals used for MHQ synthesis

Name	Chemical formula	Manufacturer	CAS	Purity
DI Water	H_2O	Inhouse source	-	
Vanillin	$C_8H_8O_3$	Jiaxing Zhonghua Chemicals	121-33-5	≥98%
Sodium hydroxide	NaOH	J.T.Baker Pellets	1310-73-2	50 wt%
Hydrogen peroxide 50%	H_2O_2	Sigma Aldrich	7722-84-1	≥99%
Sodium percarbonate	$2{\cdot}Na_2CO_3{\cdot}3H_2O_2$	Evonik Treibacher	15630-89-4	≥99%
Tetrahydrofuran	C_4H_8O	VWR Chemicals	109-99-9	≥99%
Acetic acid	$C_2H_4O_2$	Carl Roth	64-19-7	≥99%
MHQ	$C_7H_8O_3$	Sigma Aldrich	824-46-4	≥99%

1.2 Batch experiments

Reaction kinetics were investigated using a custom-made mini reactor made of stainless steel with an inner diameter of 12 mm and an inner tube height of 30 mm. The mini reactor is pictured in Figure S1. The wall thickness was 5 mm. A thermocouple (type K), installed in an 1.5 mm hole, was used to measure the temperature of the reaction mixture.

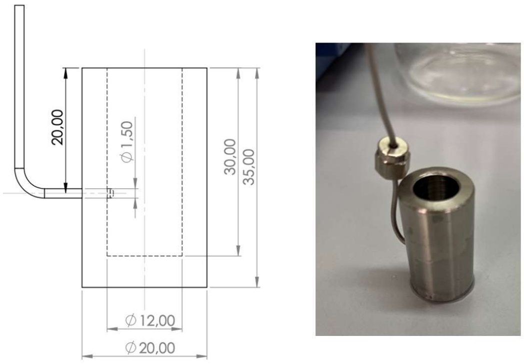

Figure S1. Custom-made temperature-controlled mini reactor for kinetic experiments.

The temperature data were recorded with a custom-built Arduino-based data acquisition (DAQ) unit. During the experiments, the reactor was placed in a temperature-controlled water bath (Lauda RC20) to maintain isothermal conditions. Additionally, the reaction mixtures were precooled to avoid a sudden temperature increase by mixing enthalpy. Intense mixing within the reactor was achieved using a small magnetic stirring bar.

The reaction requires alkaline conditions to facilitate the activity of hydrogen peroxide as a hydroperoxide anion. For batch experiments, the phase ratio of the hydrogen peroxide-containing aqueous phase to the vanillin-containing solvent phase was remained constant at 2. This optimized approach allows an increased vanillin concentration and ensures sufficient capacity of the hydrogen peroxide in the aqueous phase for highly concentrated reactions. Hydrogen peroxide is added in excess (≥ 5 equivalents) to prevent conversion limitations due to oxidant depletion.

A separate reaction was conducted for each data point, with the reaction broth quenched by acidification with acetic acid after the desired time. For the experiments, 1 mL aqueous phase containing the peroxide was mixed with 0.5 mL organic phase containing vanillin and quenched with 0.2 mL of concentrated acetic acid. pH levels were recorded in a glass beaker (containing 20 mL of the organic phase and 40 mL of the aqueous phase) under identical concentrations and temperatures. Each experiment was performed twice, and the average measured concentrations of MHQ and vanillin were used to calculate yield and conversion. The yield of the produced MHQ was calculated according to Equation 1.

$$Y = \frac{\dot{n}_{\mathrm{MHQ,produced}}}{\dot{n}_{\mathrm{Vanillin,initial}}} \qquad (1)$$

High performance liquid chromatography (HPLC) was used to measure the concentrations of vanillin and MHQ.

1.3 Reaction enthalpy

The reaction enthalpy was determined in an isolated, closed Dewar vessel. The temperature increase over time was monitored using a custom MATLAB routine in conjunction with a type K thermocouple. Continuous mixing of the reaction mixture was ensured using a laboratory stirrer (WiseStir HT 50 DX, 50–1000 rpm) operating at a low rotational speed. The experimental setup used for determination of the reaction enthalpy is shown in Figure S2.

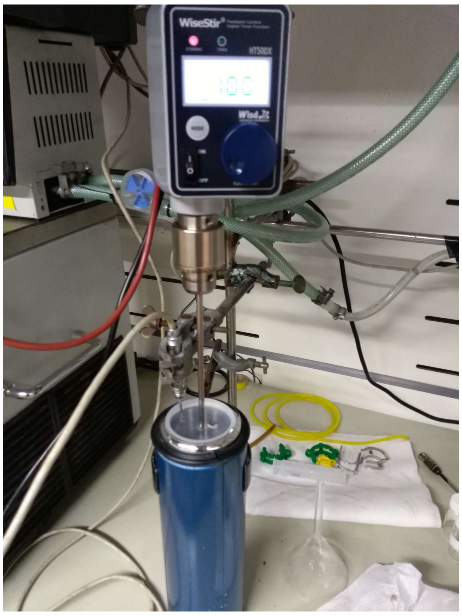

Figure S2. Experimental setup for measurement of the reaction enthalpy. An isolated Dewar vessel with laboratory stirrer is used for adiabatic reaction. The temperature sensor is connected to a computer for continuous data recording.

Two different concentrations, maintaining a constant hydrogen peroxide-to-vanillin ratio, were used for the determination of the reaction enthalpy. To prevent temperature changes caused by solvation processes, the solvent and aqueous phases were prepared outside the reaction vessel. For each measurement, 200 mL of 0.1 M NaOH solution containing 44 g L^{-1} or 88 g L^{-1} sodium percarbonate was first added to the Dewar vessel. The stirrer was then activated, followed by the addition of 100 mL of vanillin-containing THF solution (40 g L^{-1} or 80 g L^{-1}). Finally, the vessel was sealed with a plastic lid. The temperature change due to the enthalpy of mixing was considered by performing blank measurements, replicating the experiments without vanillin in the THF solution.

1.4 Continuous experiments

The geometric dimensions of the TCDC used for the continuous experiments are provided in Table S2.

Table S2. Geometric dimensions of the TCDC column

Name	Abbreviation	Value	Unit
Column diameter	D_c	0.05	m
Shaft diameter	d_{Sh}	0.025	m
Column height (active length)	H	1	m
Compartment height	H_c	0.025	m
Number of compartments	N	33	-
Rotor disc diameter	d_R	0.043	m

Column volume	V_C	2.522	L

Two individual gear pumps (Ismatec – Reglo Z with MICROPUMP GJ-N21.FF2S.B pump head for the aqueous phase and GA-X21.CFS.B pump head for the solvent phase) are installed to maintain a constant flow rate of both phases. The temperature of the reaction mixture is measured before and after the active mixing height using a thermocouple type K (TI 1 and TI 2 in Figure 4 in the main paper). A double-jacketed glass column connected to a heat exchanger (MGW Lauda - Kryomat RUK 40 S) is used to provide adequate cooling (W1 in Figure 4 in the main paper). The rotor is powered by a laboratory stirrer (WiseStir HT 50 DX, 50-1000 rpm). The control valve V3 is used to adjust the flow rate of the heavy aqueous outlet and to adjust the phase interface in the top settling zone. V4 remains permanently open to ensure an unhindered flow of the product-containing solvent phase. The separation of the solvent and aqueous phases at a specific column height is shown in Figure S3. The solvent phase sediments at the top of the column, forming a phase interface with the aqueous phase in the settling zone.

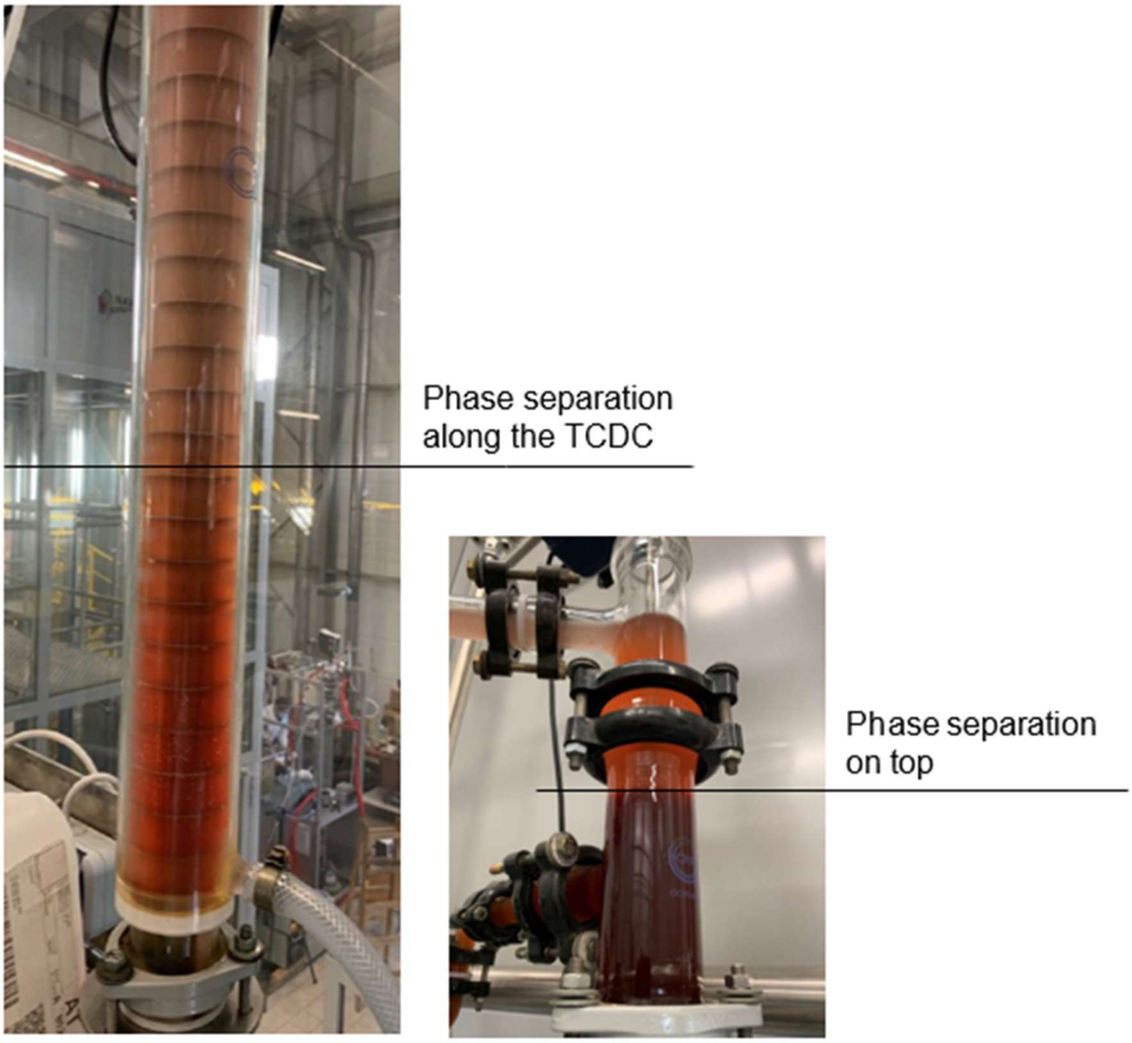

Figure S3. Phase separation along the column height of the TCDC and phase separation of the aqueous and solvent phase at the top of the column.

The water content in the solvent phase was measured using the Karl Fischer titrator TitroLine 7500 KF 05. Water removal was performed by evaporation after solvent recycling via distillation. The remaining product, a dark brown liquid, was placed in crystallization beakers and stored in a vacuum drying oven at 50°C and 100 mbar for two days. The resulting yellow crystals are shown in Figure S4.

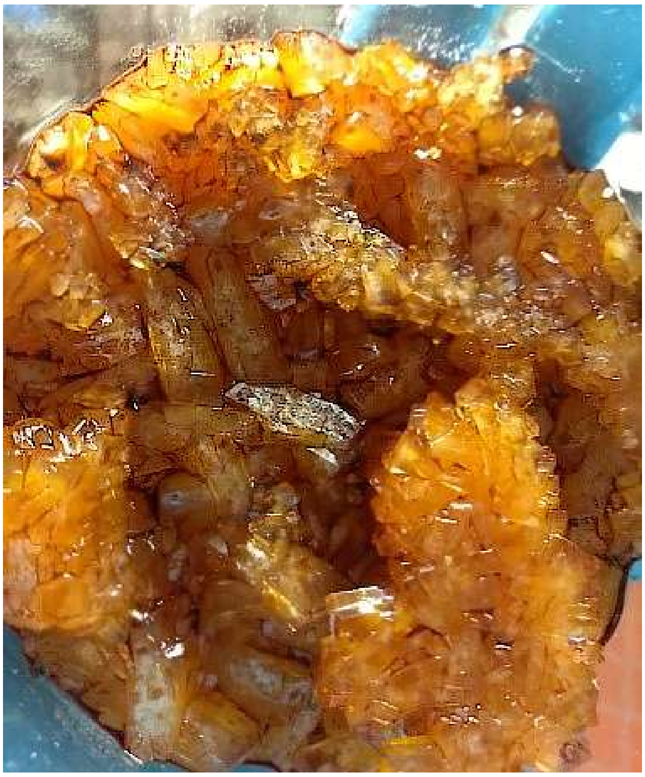

Figure S4. MHQ crystals after crystallization in vacuum oven at 50°C and 100 mbar for 2 days.

1.5 Residence time distribution

The residence time distribution (RTD) of the TCDC column was investigated to quantify deviations from ideal plug-flow behavior needed for column design. RTD experiments were conducted by adding a pulse of saturated sodium chloride solution at the bottom of the column and measuring the conductivity changes at the end of the active mixing zone. These experiments were carried out under non-reacting conditions, using deionized water as the continuous phase. The effects of rotational speed and volumetric flow rates on RTD were evaluated. Conductivity data recorded during the experiments were normalized and analyzed using the continuous stirred tank reactor (CSTR) cascade model. The number of corresponding vessels (N_{CSTR}) in series was calculated based on the maximum of the dimensionless exit age distribution ($E_{\theta,\mathrm{max}}$), as shown in Equation 2. Further analysis included the calculation of the Bodenstein number Bo (see Equation 4), and the axial dispersion coefficient D_{ax} (see Equation 3), using the dispersion model with open-open vessel boundary conditions (oo), as described by Levenspiel [17].

$$E_{\theta,\mathrm{max}} = \frac{N_{\mathrm{CSTR}} \cdot (N_{\mathrm{CSTR}}-1)^{(N_{\mathrm{CSTR}}-1)}}{(N_{\mathrm{CSTR}}-1)!} \cdot \mathrm{e}^{-(N_{\mathrm{CSTR}}-1)} \qquad (2)$$

$$E_{\theta,oo} = \frac{1}{2}\frac{1}{\sqrt{\pi\cdot\left(\frac{D_{ax}}{u\cdot L}\right)}}\exp\left[-\frac{\bar{t}\cdot\left(1-\frac{t}{\bar{t}}\right)^2}{4\cdot t\cdot\left(\frac{D_{ax}}{u\cdot L}\right)}\right] \quad (3)$$

$$Bo = \frac{u\cdot L}{D_{ax}} \quad (4)$$

In these equations, u represents the flow velocity, L the characteristic length, t the time and $\bar{t}$ the mean residence time. The mean residence time ($\bar{t}$) was calculated as a function of the total liquid phase flow rate ($\dot{V}_L$) using Equation 5. The hydraulic residence time (HRT) was then determined based on the reactor volume ($V_{Reactor}$) using Equation 6.

$$\bar{t} = 7711.72 \cdot \dot{V}_L^{-1} \quad (5)$$

$$\mathrm{HRT} = \frac{V_{Reactor}}{\dot{V}_L} \quad (6)$$

1.6 High performance liquid chromatography

High performance liquid chromatography (HPLC) measurements were performed using a SHIMADZU UFLC system equipped with a thermostatically controlled column oven. The separation of the compounds was done using a C-18 reversed-phase column of the type GEMINI® 5u C18, 4.6 × 150 mm, 5.0 µm by PHENOMENEX. Detection of the substances was carried out with a SHIMADZU SPD-20A Diode Array Detector at a wave-length of λ = 254 nm. Methanol and 0.01 M phosphoric acid in water were used as eluents. In this HPLC analysis, a linear gradient elution was used to achieve effective separation of the analytes. The elution ramp increased the methanol concentration from 2% in water to 20% in water over a span of 0–22 minutes, followed by a return to 2% methanol in water throughout the 30-minute run. The flow rate was set at 0.70 mL min^{-1}, and the column temperature was maintained at 40°C throughout the analysis.

1.7 Nuclear Magnetic Resonance

The crystalline product was analyzed by Nuclear Magnetic Resonance (NMR) and compared with a purchased reference sample as listed in Table S1. A Bruker 300 MHz NMR instrument was used to acquire ^{1}H and ^{13}C spectra. For the analysis, 20 mg of the sample was dissolved in DMSO-d6 containing, the signals were referenced by the DMSO signal at 2.50 ppm (1H) and 39.52 ppm (13C). The spectra were averaged over 16 scans with a 1-second delay, and the data were processed using Mestrenova software from Mestrelab.
